# Using Heart Rate Variability to Assess Nurses’ Stress During the COVID-19 Pandemic

**DOI:** 10.1177/01939459241252078

**Published:** 2024-05-09

**Authors:** Hanna Krieger, Cosima Rhein, Eva Morawa, Werner Adler, Jan Steffan, Nadine Lang-Richter, Matthias Struck, Yesim Erim, Marietta Lieb

**Affiliations:** 1 Department of Psychosomatic Medicine and Psychotherapy, University Hospital of Erlangen, Friedrich-Alexander University Erlangen-Nürnberg (FAU), Erlangen, Germany; 2 Department of Medical Informatics, Biometry and Epidemiology, Friedrich Alexander University of Erlangen-Nuremberg, Erlangen, Germany; 3 Group of Medical Data Analytics, Fraunhofer IIS, Erlangen, Germany; 4 Center for Sensor Technology and Digital Medicine, Fraunhofer IIS, Erlangen, Germany

**Keywords:** COVID-19, heart rate variability, nurses, recovery effect, stress

## Abstract

**Objective:**

This study aimed to assess subjective and objective parameters of stress among nurses during the COVID-19 pandemic and to examine the recovery effect of a day off.

**Methods:**

In this prospective observational trial, we measured heart rate variability (using a wearable device) and perceived stress levels on 3 working days and 1 day off. We obtained the following data using an online questionnaire: working conditions, COVID-19-related problems, depression (Patient Health Questionnaire-9), anxiety (Generalized Anxiety Disorder-7), effort-reward imbalance, and work-family conflict in a sample of German nurses (N = 41).

**Results:**

When comparing working days with a day off, we observed a significant difference for physical load (Cohen’s *d* = 0.798, *P* < .001), mental load (Cohen’s *d* = 0.660, *P* = .001), emotional exhaustion (Cohen’s *d* = 0.945, *P* < .001), and overburdening (Cohen’s *d* = 0.585, *P* = .002) with higher scores on working days. Regarding heart rate variability, we did not find a difference. Correlational analyses revealed a significant association between being afraid to get infected with COVID-19 and lower heart rate variability (*r* = −0.336, *P* = .045) and between being afraid to infect relatives and lower heart rate variability (*r* = −0.442, *P* = .007). Furthermore, a higher total sum score of work-family conflict was significantly associated with lower heart rate variability (*r* = −0.424, *P* = .01).

**Conclusion:**

As heart rate variability observations were different from those regarding subjectively perceived stress, further studies are needed to evaluate and differentiate the influence of work stress and other types of stress on heart rate variability.

The COVID-19 pandemic has placed high demands on the health care system as well as every health care worker (HCW). Several studies conducted during the COVID-19 pandemic indicated enormous physical and mental burden on hospital staff.^1-4^ The work of health care professionals can be physically and emotionally exhausting.^5^ Such high levels of workplace stress can cause depression, anxiety, insomnia, burnout, low self-esteem, and other mental health problems if left untreated.^6^ The COVID-19 pandemic has exacerbated this psychological discomfort, work stress, and emotional exhaustion among nursing staff and has led to an increased turnover intention.^5,7,8^ In particular, the type of job and direct contact with infected patients seem to be associated with higher levels of psychological distress, especially anxiety, depressive symptoms, and sleep problems.^2,3,9^ Gago-Valiente et al^5^ concluded that nurses exposed to SARS-CoV-2 in their work environment have a poorer state of mental health (higher emotional exhaustion, higher depersonalization, higher probability of nonpsychotic psychiatric diseases) than professionals without direct contact. Furthermore, it was also found that it was not the fear of getting infected themselves that led to increased perceived stress, but the possible lack of protective clothing, too little or no rest, and a possible inability to respond to patients’ worries.^1^

## Increased Mental and Physical Load of Health Care Workers in Germany due to COVID-19

Data generated by the German VOICE study group^4,10-15^ also showed that HCWs face several pandemic stressors such as fear of infecting themselves or others, increased workload, and moral distress. The authors found that various psychosocial resources such as resilience and social support were associated with fewer mental health problems among HCWs.^4^ Overall, HCWs showed significantly higher levels of anxiety and depression when compared to pre-pandemic data from the general German population^4^ and a generally increased self-reported burden.^13^ In particular, they revealed that insufficient recovery in leisure time, less trust in colleagues in difficult situations at work, and higher alcohol consumption were associated with increased depressive symptoms.^4,10^ Furthermore, increased fear of suffering from COVID-19 was positively related to anxiety symptoms^10^ and moral distress.^11^ A higher sense of coherence,^12^ elevated levels of perceived social support, and optimism were negatively associated with symptoms of anxiety and depression.^14^ The greatest stressors during the COVID-19 pandemic were the fear of infecting loved ones and family, followed by physical or mental exhaustion and change in tasks.^15^

## Heart Rate Variability as a Stress Indicator

The relationship between the autonomic nervous system and human behavior has been of great interest for a long time. Polyvagal theory, for example, has long focused on the way the human body reacts to stress.^16^ It is well known that a prolonged and/or inadequate response to stressors can lead to endocrine, metabolic, autoimmune, and psychiatric disorders.^17^ It is therefore very important to identify stress before it becomes a chronic stressor. Stress can be detected on a biological level by different parameters. One stress axis is the autonomic nervous system, for which heart rate variability (HRV) is an indicator. Several studies have already shown that HRV can be used as a valid stress indicator.^6,18-21^ HRV is defined as the fluctuation in the time intervals between consecutive heartbeats (RR intervals).^22,23^ It reflects the ability of body to respond to environmental and physiological challenges and to regulate emotions.^24^ It is a quantitative marker of the autonomic nervous system which is influenced by both the sympathetic and the vagal parasympathetic nervous system activity. These sympathetic and parasympathetic innervations to the heart cause electrical impulses that stimulate cardiac contraction.^18^ Low vagal tone is associated with poor emotion regulation, high stress, decreased stress response, and increased stress vulnerability.^25^ The parasympathetic activity regularly sends inhibitory signals that result in a temporary reduction of the heart rate. When these parasympathetic innervations are attenuated during stress, it results in lower HRV. Low HRV is therefore a stress indicator, as it reflects stress-induced changes in the autonomic nervous system.^18,26^ In addition, decreased HRV has been found in several cardiac and noncardiac disorders and can be used as a predictor of increased mortality and morbidity.^22,26-28^ Other studies also showed that higher stress is associated with lower HRV.^24^ In comparison, higher resting HRV is associated with better cognitive flexibility and executive function.^28^ Li et al^6^ were among the first to attempt to measure stress levels in nurses under real-world work conditions and to explore how HRV is correlated with their subjective stress response, workload, and work shift. They measured lower HRV during work compared with leisure time, and lower HRV was associated with more signs of social phobia and more fatigue. Jarvelin-Pasanen et al^23^ consider HRV analysis as a possible informative marker for physiological effects of workplace stressors.

## Purpose

Given these relationships, we aimed to explore physical and mental stress as subjective stress parameters in nurses during the COVID-19 pandemic as well as HRV as an objective stress indicator. Our first research question was: “Does heart rate variability correlate with sociodemographic data, working conditions, COVID-19 related-problems and psychometric measures?”. The second research question was: “How do heart rate variability and psychometric measures change on a day off compared to working days?”. The third research question was as follows: “Is there a difference in terms of the recovery effect of a day off on a biological and subjective level?”. The goal of our study was to establish a method to monitor stress load in nurses and to examine a recovery effect on a day off.

## Methods

### Study Design, Participants, and Procedures

Our study is an extension of the VOICE study,^4,10-15^ a collaboration between 5 German university hospitals (Psychosomatic departments in Erlangen, Bonn, Ulm, Cologne, and Dresden) on mental health of medical staff during the COVID-19 pandemic. We conducted a prospective observational trial and the study followed a naturalistic design to obtain data in an everyday setting for more generalizable results.

The present study was reviewed and approved by the Ethics Committee of the Medical Faculty of the Friedrich-Alexander-University Erlangen-Nürnberg (Nr. 510_20 B). All participants provided their written informed consent to participate in this study and all procedures were conducted in accordance with the STROBE Guidelines. Data, analytic methods, and study materials can be made available to other researchers on request.

The participants were nurses from 3 German hospitals. Recruitment took place at the University Hospital of Erlangen, at a hospital with geriatric and orthopedic specialization in Erlangen, and at a general hospital in Ebermannstadt. Inclusion criteria were a minimum age of 18 years old, working as a nurse in direct patient care, had sufficient German language skills, and provided written informed consent. Exclusion criteria were factors that influence the heart rate: the use of beta-blockers, antiarrhythmic drugs, or benzodiazepines; pregnancy; and breastfeeding. Participants were recruited by using hospital intranet advertising, flyers, and personal recruitment on the individual wards of the 3 hospitals. Data were collected from April to December 2021. Participants provided written informed consent during a 1-time on-site appointment. They got a weblink for an online questionnaire (online survey tool www.unipark.com) on demographic data, working conditions, COVID-19-related questions, subjectively perceived stress experience, depression, and anxiety, which was to be completed at home before starting the study. Furthermore, they were given a stress diary, which had to be completed 3 times a day (morning, noon, and evening) during the 4 study days and they received shirts for measuring HRV (24-hour measurement). We assessed 3 working days and 1 day off.

### Measures

#### Heart Rate Variability

For measuring HRV, we used shirts with integrated ECG electrodes from the company ambiotex© (Schönefeld, Germany).^29^ These shirts were connected to a mobile application via Bluetooth. We did a 24-hour HRV recording, which is the gold standard for clinical assessment of HRV, to monitor circadian rhythms^22^ and to detect interactions between situations, thoughts, feelings, and bodily reactions.^24^ Furthermore, 24-hour recording has a greater predictive power than short-term measurements.^22^ As a measure of HRV, we used the root mean square of successive differences (RMSSD), which is the square root of the consecutive time differences between normal heartbeats. First, each consecutive time difference between heartbeats is calculated in milliseconds. Then, each of the values is squared and the result is averaged before the square root of the total is determined.^22^ The RMSSD primarily reflects parasympathetic activation of the autonomic nervous system^23^ and is the preferred time-domain measure used to estimate the vagally mediated changes.^22,30^ Previous studies found that RMSSD was significantly decreased in specific experimental tasks for stress anticipation.^31^ Increased occupational stress was found to be associated with decreased HRV, especially decreased activation of the parasympathetic nervous system, as shown by a decrease in RMSSD.^23^

#### Stress Diary

For every measurement point, the following constructs were rated on a 0 to 10 Visual Analog Scale: physical load (“How much physical stress are you currently experiencing?”), mental load (“How much mental stress are you currently experiencing?”), emotional exhaustion (“How exhausted do you feel right now?”), pain (“Do you have any pain right now? How severe is it?”), and overburdening (“Do you feel overburdened right now? How much?”). In addition, in case of specific stress events during the day, participants had the possibility to indicate these stress factors in the diary.

#### Psychometric Measures

The online questionnaire consisted of the following sociodemographic data: age, gender, having children (or not), and living alone (or not). The following occupational characteristics were assessed: working full time (or part time) and occupancy level of the wards (rated on a scale from 1 “strongly below average” to 5 “strongly above average” with regard to the past 2 weeks).

The working conditions “I can recover sufficiently in my time off” (working condition 1) and “There is enough protective clothing for medical staff” (working condition 2), the 2 potential COVID-19-related problems “I was afraid to get infected” (Problem 1) and “I was afraid of infecting relatives” (Problem 2), and the 2 items of a potential work-family conflict^32^ (“The demands of my work collide with my personal and family life”; “My workload makes it difficult to fulfill my family responsibilities”) were assessed on a scale from 1 “strongly disagree” to 5 “strongly agree.”

Depressive symptoms were measured with the Patient Health Questionnaire 9 (PHQ-9).^33^ The PHQ-9 score can range from 0 to 27, since each of the 9 items can be scored from 0 (not at all) to 3 (nearly every day). Anxiety symptoms were assessed with the Generalized Anxiety Disorder 7 (GAD-7).^34^ The GAD-7 score can range from 0 to 21. A cutoff value of ≥10 has been suggested to detect probable cases of clinically significant levels of both depressive and anxiety symptoms.^33,34^ Effort and reward were assessed by 10 items of the effort-reward imbalance scale^35^ (eg, “I have constant time pressure due to a heavy work load” [effort] and “I receive the respect I deserve from my superior or a respective relevant person” [reward]), each rated on a scale from 1 “strongly disagree” to 4 “strongly agree.” In our calculations, we used the total sum score of effort and reward. To show a potential effort-reward imbalance, we calculated the ER ratio (E/(R*C)) between the sum score of effort (E) and reward (R) with the formula C (3/7 = 0.4286) to account for the unequal number of items.^35,36^ A ratio >1 indicates that the spent efforts exceed the received rewards.

### Statistical Analysis

Statistical analyses were performed with SPSS for Windows, version 28, and the program R, version 4.2.2. Statistical tests were based on an alpha level of significance of 0.05. Descriptive statistics (absolute and relative frequencies) were computed for sociodemographic, occupational, and COVID-19-related characteristics and the frequency of probable depression and anxiety. For the correlation of HRV with subjective parameters of stress, sociodemographic characteristics, occupational characteristics, COVID-19-related problems, anxiety, and depression, bivariate correlation analyses were conducted. Pearson correlation coefficients are interpreted according to Cohen: (*r* > 0.10 = small, *r* > 0.30 = moderate, *r* > 0.50 = large effect).^37^

To examine the associations among the 5 items of the stress diary with HRV and to investigate age and gender as possible confounders,^38,39^ multiple linear regression analyses were performed for the total sample. Paired *t* tests were conducted to examine the difference between a working day and a day off with regard to the subjective variables from the stress diary and the HRV. The effect size Cohen’s *d* is also reported (*d* ≥ 0.2 = small, d ≥ 0.5 = medium, and *d* ≥ 0.8 = large effect).^37^

To compute representative values for each study day that take account of the times when the stress diary was completed, we calculated the areas under the curve of the diary entries and their mean values. In case of interrupted or defective HRV measures, missing time segments were cut out of the record. Due to the partial lack of data during the day, we calculated the mean value of the logarithmized HRV over the 24-hour recording period for the 3 working days and for the day off: mean (log HRV). Due to the exploratory character of this study, we did not impute missing data.

Data were processed using a series of Python scripts created specifically for the dataset. The procedure for processing the HRV data was as follows: The raw data (RR intervals) of the shirts were imported. Incorrect sections of the signal (noise, movement artifacts, sensor failures) were marked of. The time curve of the heart rate and the heart rate variability (RMSSD) were calculated, excluding the erroneous areas. Then we performed a segmentation of the data by time of day (morning, midday, evening, night) and type of day (working days 1-3, day off). Afterward we calculated the mean values over these time periods.

Heart rate and HRV are by definition nonnegative variables (positive or zero) and therefore, strictly considered, cannot follow a Gaussian distribution. Their histograms are much better modeled by a log-normal distribution. By using a logarithm function, they can be transformed into normally distributed variables. This step is important because most of the methods used in statistical evaluations (mean values, correlations) do not deal well with the extreme outliers that such log-normal distributions have.

## Results

### Descriptive Statistics

#### Participants

Forty-one nurses participated (age: 18-63 years), 25 (61.0%) of whom were female, 17 (41.5%) had children, and 12 (29.3%) were living alone. Twenty-four (58.5%) participants worked full time, 14 (34.1%) were working in intensive care units, and the mean indicated occupancy level of the wards was 3.7 (SD: 1.0). Twenty-five participants (61.0%) indicated the occupancy level of the wards as above average (occupancy level > 3). The mean score for working condition 1 (sufficient recovery) was 2.5 (SD: 1.1) and 8 participants (19.5%) reported they could recover sufficiently in leisure time (working condition 1 score > 3). The mean score for working condition 2 (enough protective clothing) was 4.4 (SD: 0.9) and 39 participants (95.1%) agreed/were neutral (working condition 2 score > 2). The COVID-19-related problem 1 (afraid of getting infected) had mean score of 2.0 (SD: 1.0) and the mean score for problem 2 (afraid of infecting relatives) was 2.8 (SD: 1.3). Four participants (9.8 %) were afraid of getting infected and 13 (31.7%) were afraid of infecting relatives.

#### Psychometric Measures

The mean PHQ-9 score of our sample was 7.8 (SD: 4.3) and the mean GAD-7 score was 7.6 (SD: 4.1). The rate of participants with clinically significant levels of depressive symptoms was 36.6%, and the rate was 31.7% for anxiety. We measured 6.4 (SD: 2.0) as the mean work-family conflict score. The mean score for effort was 9.4 (SD: 1.7), and 15.8 (SD: 2.8) for reward. A total of 85.4% of the participants had an effort-reward imbalance ratio (E/[R*C]) > 1 and the mean ratio was 1.5 (SD: 0.5). The perceived efforts were higher than the perceived rewards.

#### Heart Rate Variability

The mean value of HRV of all participants in milliseconds was 38.3 (SD: 13.8). Table 1 shows age-dependent effects on HRV on a working day compared to a day off. Age- and gender-related differences can also be seen in Table 1: the mean HRV, averaged over the 4 study days, decreases with higher age, but a gender effect on HRV could not be detected.

**Table 1.** Heart Rate Variability (HRV): Age- and Gender-Related Differences (N = 41).

a) Age-related differences					
Age (years)	n	HRV (ms), mean (SD)		log HRV, mean (SD)	
		Working day	Day off	Working day	Day off
18-30	13	42.358 (11.131)	41.581 (11.042)	3.665 (0.267)	3.637 (0.234)
31-40	10	47.125 (19.858)	37.476 (7.586)	3.718 (0.33)	3.574 (0.203)
41-50	11	30.134 (9.004)	30.515 (11.026)	3.296 (0.267)	3.309 (0.399)
51-60	5	30.101 (20.571)	45.1 (12.988)	3.225 (0.646)	3.779 (0.293)
61-70	2	40.167 (18.876)	92.419 (95.181)	3.562 (0.448)	4.148 (1.309)
b) Gender-related differences					
Age (years), gender				HRV (ms), mean (SD)	
18-40, female				42.999 (11.621)	
18-40, male				45.122 (13.98)	
>40, female				34.585 (15.197)	
>40, male				26.808 (6.404)	
We averaged the 4 study days Measure of HRV: RMSSD (= root mean square of successive differences)					

### Correlational Analyses: Associations of HRV With Sociodemographic Data, Working Conditions, COVID-19-Related Problems, and Psychometric Measures

When referring to HRV in the following, we are referring to the mean RMSSD over all study days (including working days and days off).

Higher age was significantly correlated to lower HRV (*r* = −0.350, *P* = .04, moderate effect). A significant association was also observed between having children and lower HRV (*r* = −0.537, *P* = .001, large effect); parents had lower HRV. Furthermore, there was a significant association between being afraid to get infected (COVID-19-related problem 1) and HRV (*r* = −0.336, *P* = .045, moderate effect). Higher anxiety about getting infected was related to lower HRV. The relationship between being afraid to infect relatives and HRV (COVID-19-related problem 2) was also significant (*r* = −0.442, *P* = .007, moderate effect). Higher anxiety about infecting relatives was associated with lower HRV. Moreover, a higher total sum score of work-family conflict was significantly associated with lower HRV (*r* = −0.424, *P* = .01, moderate effect). The total sum scores for depression (PHQ-9), anxiety (GAD-7), and effort-reward imbalance were not correlated to HRV (*P* > .10, small effect sizes). Moreover, we did not find a significant correlation between working condition 1 (“I can recover sufficiently in my time off”) and HRV (*r* = 0.212, *P* = .22, small effect). The correlation between working full time and HRV was not found to be significantly associated (*r* = 0.285, *P* = .09, small effect), but showed a statistical trend (working full time being associated with higher HRV). Furthermore, the correlation between living alone and HRV (*r* = 0.321, *P* = .06, moderate effect) also showed a statistical trend (living alone being associated with higher HRV), which did not reach significance though, possibly due to limited power.

### Multivariate Analysis

When we examined the associations among the 5 items of the stress diary and HRV with age and gender as possible confounders for HRV,^38,39^ we found no significant association between physical load, mental load, emotional exhaustion, pain, and overburdening with HRV in a linear regression analysis (Table 2).

**Table 2.** Linear Regression Analyses With Log HRV as Dependent Variable (N = 41).

Stress diary item	Regression coefficient* (95% CI)	*P* value
Physical load		
Intercept	3.678 (3.351 to 4.005)	<.001
Physical load	−0.015 (−0.130 to 0.099)	.78
Age (> 40 years)	−0.342 (−0.567 to −0.118)	.004
Gender (female)	0.057 (−0.182 to 0.296)	.63
Mental load		
Intercept	3.804 (3.480 to 4.128)	<.001
Mental load	−0.062 (−0.156 to 0.032)	.19
Age (> 40 years)	−0.341 (−0.555 to −0.126)	.003
Gender (female)	0.077 (−0.157 to 0.311)	.51
Emotional exhaustion		
Intercept	3.694 (3.352 to 4.035)	<.001
Emotional exhaustion	−0.016 (−0.102 to 0.070)	.71
Age (> 40 years)	−0.347 (−0.567 to −0.128)	.003
Gender (female)	0.065 (−0.180 to 0.309)	.59
Pain		
Intercept	3.648 (3.424 to 3.872)	<.001
Pain	−0.007 (−0.087 to 0.073)	.86
Age (> 40 years)	−0.343 (−0.574 to −0.112)	.005
Gender (female)	0.060 (−0.189 to 0.310)	.63
Overburdening		
Intercept	3.644 (3.396 to 3.892)	<.001
Overburdening	0.002 (−0.131 to 0.134)	.98
Age (> 40 years)	−0.349 (−0.571 to −0.128)	.003
Gender (female)	0.053 (−0.195 to 0.301)	.67

* Unstandardized beta weights.

### Differences in HRV and Psychometric Measures Between Working Day and Day Off

We observed higher values in all 5 items of our stress diary on a working day compared to a day off (Table 3). A significant difference with large effect size (Cohen’s *d* = 0.945, *P* < .001) was found for emotional exhaustion and with medium effect sizes for the items physical load (Cohen’s *d* = 0.798, *P* < .001), mental load (Cohen’s *d* = 0.660, *P* < .01), and overburdening (Cohen’s *d* = 0.585, *P* < .01). Regarding HRV, we did not find a significant difference between a working day and a day off (*P* = .50).

**Table 3.** Difference Between a Working Day and a Day Off (N = 41).

Measures	Test statistic (working day)	Test statistic (day off)	Effect size	*P* value
a) Subjective perception (stress diary)				
Physical load	2.753	1.719	0.798	<.001***
Mental load	3.108	2.042	0.660	.001**
Emotional exhaustion	3.874	2.381	0.945	<.001***
Pain	1.514	1.328	0.036	.84
Overburdening	1.531	0.811	0.585	.002**
b) Heart rate variability				
Mean (log HRV)	3.510	3.585	0.125	.50

Test statistics: *t* test (*t* value), effect size: Cohen’s *d*. We averaged the 3 working days.

Abbreviation: HRV, heart rate variability.

* *P* < .05. **P < .01. ***P < .001.

## Discussion

The aim of this study was to assess subjective and objective parameters of stress among German nurses and to examine the recovery effect of a day off during the COVID-19 pandemic.

Similar to the study of Chen et al,^1^ the fear of getting infected with the corona virus was not often experienced in our study population. Only 9.8% of our participants were afraid of getting infected. Unlike the study of Chen et al,^1^ the lack of protective clothing was no longer an important stressor for our participants. Just 4.9% stated that there was not enough protective clothing. This is probably due to the fact that our survey was conducted at a later time point where the shortage of protective clothing was not a problem as it was at the beginning of the pandemic. As shown in the VOICE study,^4,10-15^ the increased workload (61.0% of our participants reported the occupancy rate of the wards as above average) and the fear of infecting relatives (31.7%) were still relevant pandemic stressors in our survey.

In our study, 80.5% of the nurses could not recover sufficiently in their leisure time. However, we could not find any significant correlation between the item “I can recover sufficiently in my time off” and HRV (*r* = 0.212, *P* = .22), possibly due to low statistical power. Nevertheless, we consider insufficient recovery in leisure time to be an important stressor and this demonstrates how important it is to ensure recovery from work to assure well-being for nurses.

Furthermore, in our sample, 36.6% of the participants showed clinically significant levels of depression and the rate of clinically significant levels of anxiety was 31.7%. These are higher percentages than in previous studies: Morawa et al^10^ found clinically significant depressive symptoms in 21.6% of nurses and clinically significant levels of anxiety in 19.0% during the first wave of COVID-19. Benke et al^40^ investigated a representative sample of the German adult population before and during the COVID-19 pandemic and found an increase in clinically significant depressive symptoms (9.7% vs 12.0%), as well as in clinically significant anxiety symptoms (6.8% vs 7.9%) during the pandemic. This dynamic could be explained by the fact that our survey took place at a later point in the pandemic, when people may have been even more burned out. Another explanation could be our smaller sample size.

Considering the mean values of HRV in our sample, they were within the 25th to 50th percentile of the age-specific norms.^41^ Thus, in contrast to the high psychological burden, our participants seem to match the norm on the physiological level. Concerning the mean HRV, averaged over the 4 study days (Table 1), we are in line with previous results showing that HRV decreases with higher age.^38,41-43^ Unlike previous studies, we did not observe any gender-dependent changes in HRV. Voss et al^38^ found lower HRV in men, whereas Sammito et al^39^ identified higher values of HRV in men. However, different measurement methods were used, so comparisons must be made with caution.^41^

In contrast to the results of Li et al,^6^ we were not able to find a significant difference in HRV when comparing working days to a day off. Li et al^6^ used HRV analysis to examine workplace stress in nurses. They measured lower HRV during work compared with rest time. One explanation for this difference to our study could be that Li et al^6^ considered breaks during a working day as recovery and did not measure HRV on a day off, as we did. In their sample, HRV was higher during breaks than during actual work.

However, we found significantly higher levels of subjective physical load, mental load, exhaustion, and overburdening on a working day compared to a day off. Pain was also reported to be less on a day off, but not to a significant level. This may be due to the fact that pain is something more chronic, which does not habituate on a day off. Our data also show that there was no significant correlation between the 5 subjective items (physical load, mental load, emotional exhaustion, pain, overburdening) from the stress diary and HRV (Table 2). In addition, we did not find any significant correlation between depressive and fear-related symptoms and HRV.

In our study, having children, fear of infecting loved ones, work-family conflict, and higher age were significantly associated with HRV. Similarly, Jerg-Bretzke et al^15^ discovered the fear of infecting loved ones and the family as the greatest stressors associated with the COVID-19 pandemic, followed by physical or mental exhaustion and change in tasks. This is important to mention since professionals with more caregiving responsibilities at home were disproportionately affected during the pandemic.

Furthermore, our finding that working full time is associated with higher HRV seems to be contradictory at first sight. An explanation for that result could be that particularly stressed employees have already reduced their job to part time or that there are certain stress factors in leisure time. For example, in the stress diary, some participants mentioned a burden of taking care of their children on the day off or sleep problems. Others indicated leisure-time stress due to pets or medical appointments. This shows that not only can working be a source of stress, but also the individual’s lifestyle. Another explanation could be that those who transitioned to part-time work or left their jobs to care for children or other family at home had to face reduced income and financial challenges.

In summary, our analysis found a discrepancy between subjective and objective levels of stress. Our participants felt subjectively recovered on a day off, but biologically, measured by HRV, there was no difference between work and leisure time. Concerning these relations, we hypothesize that stress and exhaustion are more likely to be perceived on a psychological level. On a physical level, nurses may be more resilient to work stress. This mental stress of our sample can be seen by the higher values of the 5 stress diary items on working days and by the high rate of clinically significant depressive and anxiety symptoms. The participants seem to be quite stress-resistant on a biological level, as their HRV was in the normal range, during leisure time and working days. Several days in a row may be necessary to determine any physical effects and 1 day off may not be enough to detect effects in HRV. Another explanation for our results could be that only persistent psychological stress may have a significant effect on HRV. This argument could be strengthened by our significant correlation between having children and lower HRV (*r* = −0.537, *P* = .001, large effect), between being afraid to infect relatives and lower HRV (*r* = −0.442, *P* = .007, moderate effect) and between a higher total sum score of work-family conflict and lower HRV (*r* = −0.424, *P* = .01, moderate effect). Acute work stress varies in severity, whereas anxiety and work-family conflict may be a more long-lasting and more persistent stress factor, thus having a stronger effect on HRV.

Still, we agree with the authors of some previous studies^6,18-20,23^ who have already shown that HRV can be used as a valid stress indicator, in our case especially concerning family factors, and that HRV can be useful for early detection and prevention of stress, for example, in occupational health care. Further studies are needed in larger samples to replicate our results and to assess and differentiate stress and HRV over a longer time course and smaller time intervals.

### Strengths and Limitations

To the best of our knowledge, the present study is the first survey on stress of nurses in Germany measured by HRV. Furthermore, we compared objective and subjective parameters of stress. Nevertheless, our study has also some limitations. Since we were recruiting participants who belong to a profession that is very busy and overburdened, we were only able to collect data from a small sample, which affects the power of the study. Therefore, small effects might not have been detected. In addition, our participants were from different wards with different workloads and different numbers of consecutive workdays, as well as days off at the time of the measurements. Due to different shift schedules, we were unable to standardize the sequence of study days. It is also possible that our sample was not stressed enough to show large effects. Moreover, we measured on 3 working days, of which we have formed the mean values, and only on 1 day off. Possibly, after several successive days, we could also have identified an effect on HRV. Further studies should consider measuring several days in a row. Furthermore, our recordings were sometimes interrupted by motion artefacts and problems with the Bluetooth connection. Due to this, we could not record the complete 24 hours for all participants and had to use the mean value of the logarithmized HRV over the 24-hour recording period. Therefore, we cannot make any conclusions about slight fluctuations during the day nor nightly recovery of HRV.

### Conclusion

We consider HRV to be a possible objective stress indicator, and future research should continue to examine the practical feasibility of using HRV measurement for detecting and preventing stress and stress-related conditions in nurses. However, not all types and/or intensities of stress factors seem to be reflected in HRV. To be able to generalize future results, larger samples should be investigated and precise regulations should be made, such as how many working days or days off may lie before or between the measurements. As HRV observations were different from those regarding subjectively perceived stress, further studies are needed to evaluate and differentiate the influence of work stress and other types of stress on HRV and the feasibility of HRV as a marker of work stress.
